# Status migrainosus as an initial presentation of multiple sclerosis

**DOI:** 10.1186/s40064-015-0818-9

**Published:** 2015-01-23

**Authors:** Raed Alroughani, Samar F Ahmed, Riyadh Khan, Jasem Al-Hashel

**Affiliations:** Division of Neurology, Department of Medicine, Amiri Hospital, Arabian Gulf Street, Sharq, 13041 Kuwait; Neurology Clinic, Department of Medicine, Dasman Diabetes Institute, P.O. Box 1180, Dasman, 15462 Kuwait; Department of Neurology, Ibn Sina Hospital, P.O. Box 25427, Safat, 13115 Kuwait; Department of Neurology & Psychiatry, Al-Minia University Hospital, P.O. Box 61519, Minia, 61111 Egypt; Department of Medicine, Kuwait University, P.O. Box 24923, Safat, 13110 Kuwait

**Keywords:** Migraine, Multiple Sclerosis, MRI

## Abstract

**Background:**

Demyelinating plaques may induce headache through disruption of the pathways, which are implicated in the pathogeneses of migraine. We report a case of 25-year-old female patient, who presented with status migrainosus fulfilling the criteria of international classification of headache disorder. She was eventually diagnosed with multiple sclerosis (MS) after an extensive work-up and long-term clinical and radiological follow-up.

**Findings:**

At the onset of status migrainosus, magnetic resonance imaging (MRI) revealed the presence of several demyelinating lesions fulfilling Swanton criteria. She was started on migraine prophylactic treatment but there was no subsequent response. One year later, she presented with recurrent status migrainosus and a follow-up MRI revealed multiple gadolinium-enhancing lesions in the brain. She was treated with abortive migraine medications. Within the following 2 year, she developed ascending parasthesia and weakness of both lower limbs indicative of incomplete transverse myelitis in association with recurrent status migrainosus. A diagnosis of MS was established based on a follow-up MRI that satisfied the revised 2010 McDonald criteria. Both the headache and neurological signs improved with IV methylprednisolone therapy. Her headache entered remission after initiation of a disease modifying therapy.

**Conclusion:**

Status migrainosus can be the initial presentation of MS. Unresponsiveness to migraine prophylactic therapy in the presence of active demyelinating plaque in MRI brain may pose a diagnostic challenge and a diagnosis of MS might be considered.

## Background

Multiple sclerosis (MS) can cause various neurological symptoms depending on the location of demyelinating plaques (Putzki et al. 2009). Although the prevalence of migraine is threefold higher in MS patients compared to general population, headache is not typically considered as one of the symptoms of MS (Kister et al. 2010a; Noseworthy et al. 2000). Herein, we describe a young female patient who presented with status migrainosus as the initial presenting symptom of MS.

## Case presentation

**Figure 1:**
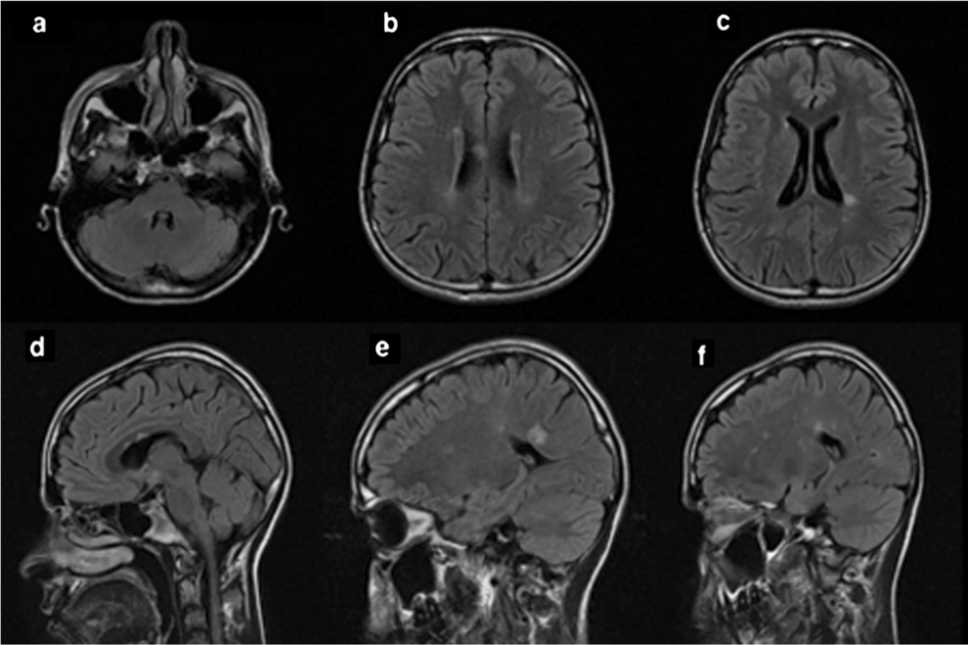
**Baseline axial and sagittal T2/flair MRI brain images (September 2011) showed demyelinating plaques involving the right middle cerebellar peduncles (a), periventricular areas (b,c) and corpus callosum (d-f).**

**Figure 2:**
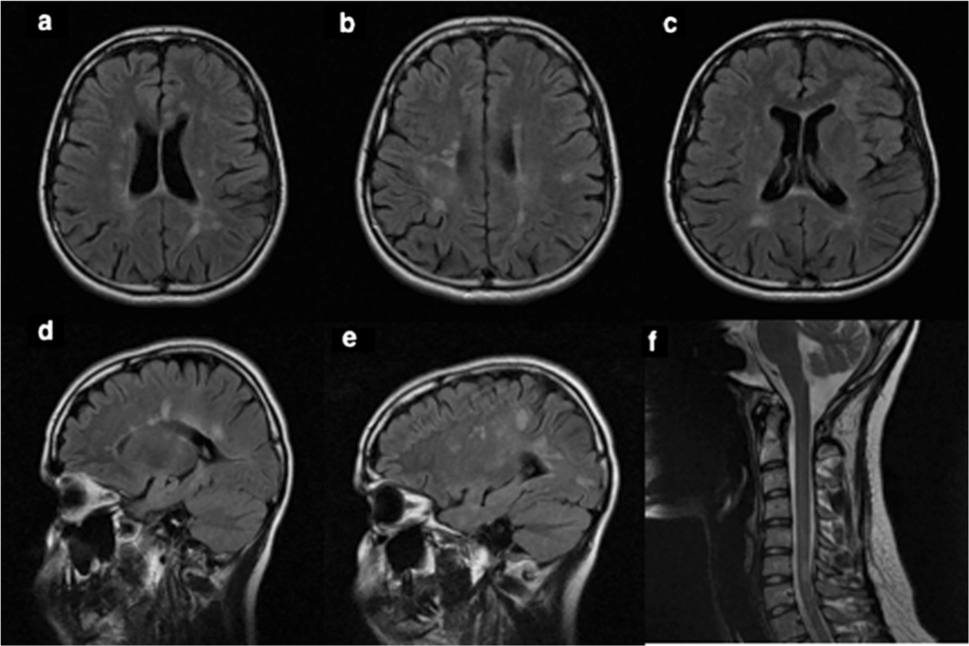
**Follow-up MRI brain images (October 2012) showed an increase in the lesion load on axial (a-c) and Sagittal (d-e) T2/flair images when compared to the baseline MRI.** Sagittal T2 spine images revealed a demyelinating plaque at C6 level **(f)**.

**Figure 3:**
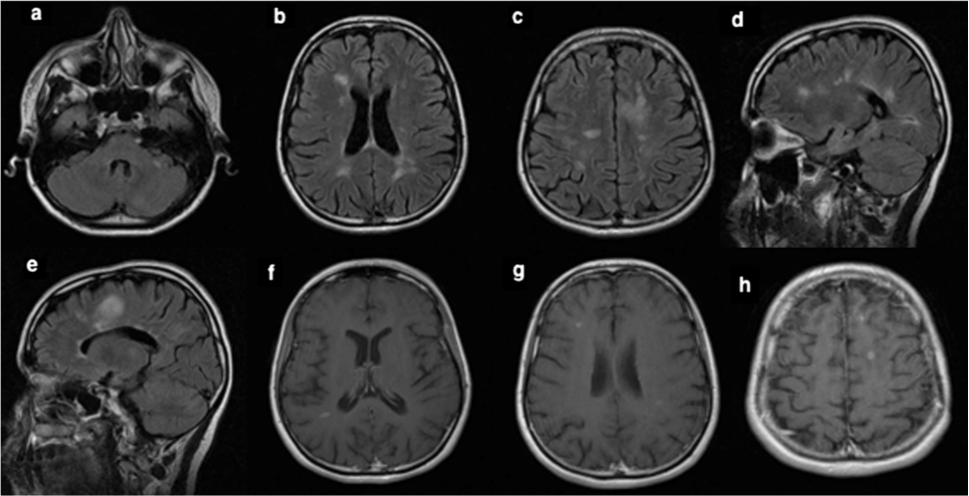
**Follow-up MRI brain scans (April 2013) showed new T2/flair lesions in periventricular and juxtacortical regions with an increase in the overall lesion load when compared to the previous MRI scan as seen in the axial (a-c) and sagittal images (d-e).** Enhanced Axial T1 MRI images showed five gadolinium-enhancing supratentorial lesions involving both hemispheres **(f-h)**.

A 25-year-old female patient presented with severe, left parieto-temporal throbbing headache associated with nausea, vomiting and photophobia, which lasted for 7 days. Although she never had a previous history of headache, she continued to have recurring migraine headaches several times per week. Her headache fulfilled the diagnostic criteria of international classification of headache disorder, third edition (ICHD-3) (Headache Classification Committee of the International Headache S 2013). Her neurologic examination was unremarkable. At the time of the initial presentation, unenhanced magnetic resonance imaging (MRI) brain showed subcortical and periventricular T2/Flair hyperintense lesions, with involvement of corpus callosum, left temporal lobe and right middle cerebellar peduncle fulfilling Swanton Criteria (Figure 1) (Swanton et al. 2006). She was diagnosed as migraine with radiologically isolated syndrome (RIS). She was started on topiramate as a prophylactic migraine therapy, which was subsequently discontinued due to poor response. She continued to use abortive medications (triptans & non-steroidal anti-inflammatory drugs “NSAIDs”) till her presentation with recurrent status migrainosus one year later. A follow-up MRI revealed multiple new T2/ flair hyperintense lesions in the brain and spinal cord indicative of radiological progression (Figure 2). A course of propranolol was initiated as alternative prophylactic migraine treatment but she continued to have recurrent severe headaches. A follow-up MRI brain with gadolinium, 6 months later, showed new T2/flair lesions in the periventricular, corpus callosal and juxtacortical areas along with new Gad-enhancing lesion at multiple supratentorial sites (Figure 3).

**Figure 4:**
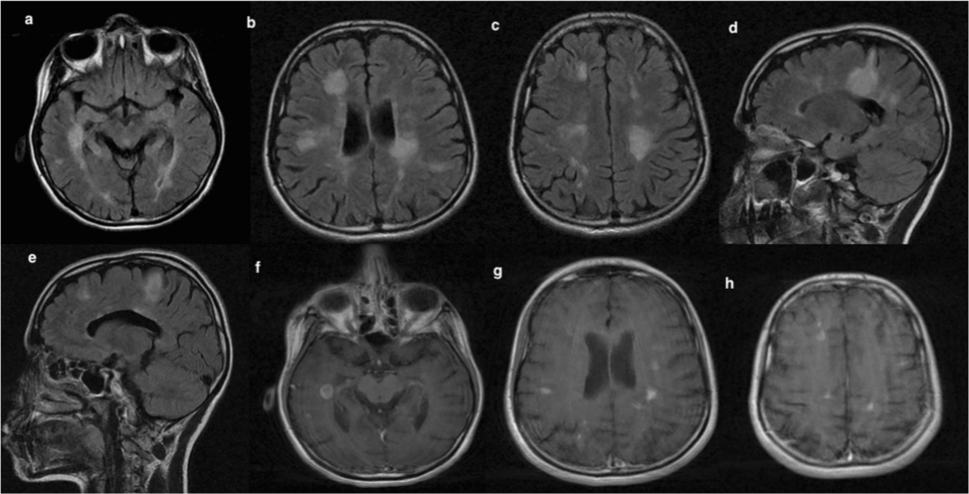
**Follow-up MRI brain scans (March 2014) showed a significant increase in the lesion load involving the periaqueductal gray matter (a), periventricular and juxtacortical regions (b,c), corpus callosum, extending lesions to cortical areas (d,e) in both axial and sagittal T2/flair scans.** Enhanced axial T1 MRI scans showed the presence of new gadolinium-enhancing lesions in the temporal, frontal and parietal lobes **(f-h)**.

Within one year, the patient presented with one-week history of numbness and weakness of both lower legs and urgency of micturition in association with an attack of status migrainosus. Neurological examination showed bilateral pyramidal weakness of lower limbs, pathological brisk deep tendon reflexes and extensor plantars along with loss of vibration and pinprick sensation up to thoracic (T12) level. A follow-up MRI brain with gadolinium revealed multiple new enhancing lesions with an open-ring pattern (Figure 4). A lumbar puncture was performed and the cerebrospinal fluid analysis revealed normal cell count, protein, and glucose. Oligoclonal bands were positive in the CSF and absent in the serum. Visual evoked potentials revealed delayed P100 wave latencies in both eye. However, somatosensory and brainstem evoked potentials were within normal limits. Autoimmune profile was unremarkable.

The patient was diagnosed with MS according to the revised 2010, McDonald criteria since she presented with incomplete transverse myelitis and after MRI showed dissemination in time and space (Polman et al. 2011). Her headache and neurological symptoms/signs improved significantly after a 5-day course of intravenous methylprednisolone. She was started on oral fingolimod 0.5 mg once daily as disease modifying therapy given her reluctance to use any injectable forms of medications. The headaches entered a remission phase for the next following months.

## Discussion

Our case illustrates the association between the onset/ recurrence of status migrainosus with progressive T2/flair and new gadolinium enhancing demyelinating lesions on the initial and subsequent MRIs (1.5 Tesla field strength). The presence of demyelinating lesions at brainstem may explain the presentation of MS onset with migraine headache (Gee et al. 2005). Periaqueductal gray matter (PAG), located in the midbrain, is thought to be a key area in the pathogenesis of migraine. PAG acts as a rely station between cortical and brainstem structures and it modulates pain by providing an antinociceptive effect to the primary afferent system as well as influencing the autonomic and behavioral responses (Raskin et al. 1987). PAG has connections to the trigeminal nucleus caudalis (TNC), which runs from the medulla down into the region of the third cervical segment where it blends gradually into the cervical dorsal horns. Some refer to this region as the trigeminocervical complex (Ward 2012). Fibers from upper cervical roots enter the TNC, which sends fibers rostrally to the thalamus and collaterals to the autonomic nuclei in the brainstem and the hypothalamus. Thalamic neurons project to the somatosensory cortex but also to areas of the limbic system (Goadsby et al. 2002). A prevailing theory in the pathogenesis of the migraine attack is that hyperexcitability develops along the trigeminovascular pathway, probably facilitated by a dysfunction of the descending pain modulatory circuits (Raskin et al. 1987; Ward 2012; Goadsby et al. 2002).

The causal relationship between migraine and MS, in our case, was suggested by the subsequent improvement of headache with a DMT. On the other hand, a response to corticosteroids such as methylprednisolone does not necessarily this causal relationship as both conditions may show improvement with the use of corticosteroids. Both migraine and MS are relatively common in young females and both may accidentally co-exist. The prevalence of migraine in patients with MS is between 20% to 45% (Kister et al. 2010b). The diagnosis of migraine precedes MS in most cases (Kister et al. 2010a; Fragoso & Brooks 2007; Yetimalar et al. 2008; Villani et al. 2008) and it is suggested to be a presenting symptom of MS (Kister et al. 2012; Zorzon et al. 2003; Lin et al. 2013). Freedman et al. reported migraine headache at MS onset in 18 (1.6%) out of 1113 patients with MS and an additional 26 (2.3%) patients experiencing recurrent headaches at the time of MS exacerbation, often accompanied by posterior fossa signs (Freedman & Gray 1989).

Migraine and MS share several features. Both have a relapsing-remitting course affecting the central nervous system with occasional chronic evolution, more prevalent in women of childbearing age, and both appear to be influenced by hormonal changes; i.e., less exacerbations throughout the first two trimesters of pregnancy (Kister et al. 2010b).

The mechanism behind the association of MS and migraine is debatable and several hypotheses were introduced. First, headache may be induced by the inflammatory demyelinating MS lesions by disrupting the pathways implicated in the pathogenesis of migraine (Kister et al. 2010b). It was observed that lesions within the midbrain especially in the PAG were commonly associated with comorbid migraine in MS patients (Gee et al. 2005; Tortorella et al. 2006). It should be noted that the inflammatory process caused by demyelination is not necessarily the cause of headaches given the incidental co-existence of both conditions. Second, a dysregulation of the serotoninergic system caused by demyelinating lesions has been linked to the headache exacerbation (Sandyk & Awerbuch 1994). Third, cortical demyelination has been shown to accelerate cortical spreading depression, which is a key mechanism in migraine pathophysiology (Merkler et al. 2009). Although cortical demyelinating plaques were seen in our case (Figure 4d,e), it is often difficult to detect definitive cortical involvement given the low sensitivity of conventional MRI. Finally, it is suggested that migraine with aura may be associated with MS activity since the development of aura involves activation of matrix metalloproteins resulting in subtle increase in permeability of the blood–brain barrier and thus presumptive neuro-inflammation (Gursoy-Ozdemir et al. 2004). Kistar et al. proposed that certain privileged central nervous system compartment to circulating T cells may be sensitized during migraine aura (Kister et al. 2010b).

## Conclusion

Status migrainosus in the presence of demyelinating MRI features at the initial presentation may pose diagnostic dilemma. Failure to respond to prophylactic migraine therapy in view of progressive demyelinating features on follow-up MRIs may point to another relatively common neurological disorder such as MS among young women.

## Consent

Written informed consent was obtained from the patient for publication of this Case report and any accompanying images. A copy of the written consent is available for review by the Editor-in-Chief of this journal.
